# Patients with psoriasis resistant to multiple biological therapies: characteristics and definition of a difficult‐to‐treat population

**DOI:** 10.1111/bjd.21048

**Published:** 2022-05-24

**Authors:** Luca Mastorino, Gabriele Roccuzzo, Paolo Dapavo, Niccolò Siliquini, Gianluca Avallone, Marco Rubatto, Martina Merli, Andrea Agostini, Simone Ribero, Pietro Quaglino

**Affiliations:** ^1^ Department of Medical Sciences, Dermatologic Clinic University of Turin Turin Italy

dear editor, Over the past two decades the proliferation of biological therapies for moderate‐to‐severe psoriasis has enabled dramatic response outcomes.^1^ As the number of molecules has increased, so has the number of patients experiencing an increasing number of biological drugs over the years, following initial therapeutic failures or secondary to acquired resistance. These patients have been defined as ‘multi‐failure patients’,^2^ yet no consensus on this definition has been reached, as this term has been invariably attributed to patients who have taken more than two, or more than four biologics, with little or no success.^1^, ^2^, ^3^

Currently, 10 biological agents are available for the treatment of psoriasis vulgaris, which act by inhibiting different targets [i.e. interleukin (IL)‐17, IL‐23, IL‐12 and tumour necrosis factor‐α] over a total of four generations of biologics. According to these considerations and in the absence of a clear guideline definition, it is our opinion that patients without response to at least four biologics should fall into the category of multi‐failure (or multi‐resistant) patients.^4^ Given the limited evidence reported for this population in the literature, we report the experience of the Dermatologic Clinic of the University of Turin.

Among 783 patients treated between November 2020 and November 2021 we identified 10 multi‐resistant patients. The patients’ characteristics are summarized in Table 1. No significant differences between these patients and the general population without psoriasis were observed for sex (50% vs. 35·2% female, *P* = 0·33), age of psoriasis onset (34 vs. 34·3 years, *P* = 0·96), psoriatic arthritis (40% vs. 29·3%, *P* = 0·46), body mass index (29 vs. 27 kg m^−2^, *P* = 0·27), cardiovascular disease (70% vs. 44%, *P* = 0·12), diabetes mellitus (20% vs. 12·7%, *P* = 0·37) or initial Psoriasis Area and Severity Index (PASI; 10·6 vs. 14·7, *P* = 0·07). The age at analysis was significantly different, respectively mean 52·3 (SD 15·2) years in patients and 63·3 (SD 4) years in the general population (*P* = 0·022). This difference may be related to the fact that older patients are more likely to experience more treatment options over time, also considering the similar age of onset.

**Table 1:** Patient characteristics, previous biological and systemic treatment, and response to the last biologic

Patient	Age (years), sex	BMI	Age of psoriasis onset (years)	Type of psoriasis	PsA	Current biological treatment	Previous biologics (duration in months)	Previous systemics	Follow‐up under biologics (months)	Baseline PASI	PASI 16 weeks	PASI 28 weeks
1	68, F	26·7	47	Vulgaris	No	Risankizumab	Adalimumab (20),^a^ etanercept (6), infliximab (84),^a^ ixekizumab (11)	Methotrexate, ciclosporin, acitretin	120	11	0	0
2	55, M	26·5	37	Vulgaris	No	Risankizumab	Adalimumab (8), secukinumab (24),^a^ ixekizumab (16),^a^ brodalumab (12)	Ciclosporin, acitretin	60	8	4	2
3	67, F	42·9	27	Vulgaris	No	Risankizumab	Adalimumab (36), infliximab (12), ixekizumab (24), brodalumab (12)	Methotrexate, ciclosporin	84	19	2	2
4	65, F	26·1	24	Vulgaris	Yes	Risankizumab	Adalimumab (39),^a^ etanercept (32),^a^ certolizumab (4), ixekizumab (2)	Methotrexate, ciclosporin, acitretin	79	15	0	0
5	66, M	32·3	46	Vulgaris	No	Risankizumab	Adalimumab (na), infliximab (na), certolizumab (na), guselkumab (na)	Methotrexate, ciclosporin	90	6	0	
6	59, M	22·6	36	Vulgaris	Yes	Guselkumab	Infliximab (na), etanercept (na), ixekizumab (na), adalimumab (na)		114	0·1	0	
7	61, M	29·7	23	Vulgaris	No	Guselkumab	Etanercept (79), efalizumab (4), adalimumab (48),^a^ secukinumab (20)^a^	Ciclosporin	151	10	3	3
8	66, F	26·3	22	Erythrodermic	No	Risankizumab	Infliximab (4), etanercept (4), adalimumab (64), ustekinumab (12), ixekizumab (28),^a^ guselkumab(13)	Methotrexate, ciclosporin, acitretin	125	7	5	5
9	63, M	26	40	Inverse	Yes	Brodalumab	Adalimumab (6),^a^ etanercept (2), infliximab (6), secukinumab (na), ixekizumab (na)	Methotrexate, ciclosporin, acitretin	102	10	3	1
10	63, F	31	38	Pustular	Yes	Secukinumab	Adalimumab (na), etanercept (na), infliximab (na), ustekinumab (70)	Methotrexate, ciclosporin	102	20	10	10

Further details on comorbidities and Psoriasis Area and Severity Index (PASI) scores beyond week 28 are available on request. BMI, body mass index (in kg m^−2^); F, female; M, male; na, not available; PsA, psoriatic arthritis. ^a^Reached ≥ 90% improvement in PASI under the treatment.

Three patients had difficult‐to‐treat variants of psoriasis (one erythrodermic, one pustular and one inverse). All 10 patients had failed at least four biologics and are currently taking an additional monoclonal antibody. Eight of these 10 patients had failed to respond to four drugs, while one patient had failed five and one had failed six. Anti‐tumour necrosis factor‐α agents were the most commonly failed biologics (10 patients failed adalimumab, seven etanercept, seven infliximab, and two certolizumab pegol). Ustekinumab failed in two patients, efalizumab in one, ixekizumab in seven, secukinumab in three and brodalumab in two. Among IL‐23 inhibitors, only guselkumab failed in this population, in two patients. As for previous nonbiological systemic therapies, seven patients had taken methotrexate, nine ciclosporin and five acitretin. Currently, six patients are taking risankizumab, two guselkumab, one brodalumab and one secukinumab.

The mean PASI at baseline of the last biological therapy was 10·2, and at 16 weeks the mean PASI dropped to 3. Three patients on risankizumab reached ≥90% improvement in PASI and all three had 100% improvement. Over the weeks, the response was maintained: at 28, 40 and 52 weeks the mean PASI was 2·8, 4 and 3, respectively. No safety concerns arose during treatment. To date, the mean follow‐up since the beginning of the biological therapy for the 10 multi‐resistant patients is 102 months.

To the best of our knowledge, we describe the first study dedicated to patients with psoriasis not responding to at least four biological treatments. Data regarding these patients are scarce in the literature, with a few good responses described with brodalumab, ixekizumab and secukinumab.^2^, ^5^, ^6^ Herein we report data regarding IL‐23 inhibitors, in particular risankizumab, which showed a remarkable response allowing 100% improvement in PASI to be reached at 16 weeks in three of six patients. Our data are in line with the findings reported by Megna *et al*. in a population of patients who had failed to respond to at least one biological treatment.^1^

As for possible predictive factors on the causes of failure and subsequent therapeutic switch, studies in the literature identify smoking, body mass index and female sex as causes.^7^ In our population, no statistically significant demographic or disease characteristics were identified, yet the low sample size limits the value of our observations. Overall, while the available therapeutic armamentarium allows a high number of therapeutic attempts, multi‐resistant patients represent an increasing challenge for the clinician. In our experience, the modern IL‐23 inhibitors have proven to be a valid therapeutic option. As the definition of predictive factors in this type of patient is difficult, and literature data are conflicting, proof‐of‐concept studies are definitely needed to better define this population, both in terms of predictive factors and in the identification of appropriate therapeutic strategies.^8^ Future guidelines should also guide parameters for therapeutic switches between the same and different classes of biological drugs.
